# Design and Optimization of Germanium-Based Gate-Metal-Core Vertical Nanowire Tunnel FET

**DOI:** 10.3390/mi10110749

**Published:** 2019-10-31

**Authors:** Won Douk Jang, Young Jun Yoon, Min Su Cho, Jun Hyeok Jung, Sang Ho Lee, Jaewon Jang, Jin-Hyuk Bae, In Man Kang

**Affiliations:** 1 School of Electronics Engineering, Kyungpook National University, Daegu 41566, Korea; wkdejr16@naver.com (W.D.J.); chominsu15@naver.com (M.S.C.); jjhyeok10@naver.com (J.H.J.); jim782jim@naver.com (S.H.L.); j1jang@knu.ac.kr (J.J.); jhbae@ee.knu.ac.kr (J.-H.B.); 2 Center for BioMicroSystems, Brain Science Institute, Korea Institute of Science and Technology (KIST), Seoul 02792, Korea; da1004rk@naver.com

**Keywords:** tunnel field-effect transistor (TFET), low power, vertical nanowire, core–shell, germanium, technology computer-aided design (TCAD)

## Abstract

In this paper, a germanium-based gate-metal-core vertical nanowire tunnel field effect transistor (VNWTFET) has been designed and optimized using the technology computer-aided design (TCAD) simulation. In the proposed structure, by locating the gate-metal as a core of the nanowire, a more extensive band-to-band tunneling (BTBT) area can be achieved compared with the conventional core–shell VNWTFETs. The channel thickness (*T*_ch_), the gate-metal height (*H*_g_), and the channel height (*H*_ch_) were considered as the design parameters for the optimization of device performances. The designed gate-metal-core VNWTFET exhibits outstanding performance, with an on-state current (*I*_on_) of 80.9 μA/μm, off-state current (*I*_off_) of 1.09 × 10^−12^ A/μm, threshold voltage (*V*_t_) of 0.21 V, and subthreshold swing (SS) of 42.8 mV/dec. Therefore, the proposed device was demonstrated to be a promising logic device for low-power applications.

## 1. Introduction

The power consumption of future transistors has become one of the most important problems in the semiconductor industry. As the device dimensions, such as the minimum feature size, are scaled down, the importance of the off-state power as well as the active power becomes significant. Particularly, low standby power and low supply voltage (*V*_DD_) operation are necessary in various electronics applications, such as mobile devices, wearable devices, and internet-of-things (IoT) systems [1,2,3]. Considering these aspects, the tunnel field-effect transistor (TFET) is one of the most promising logic devices. TFETs have advantages such as low off-state current (*I*_off_), low subthreshold swing (SS), and low power consumption compared with the conventional metal-oxide-semiconductor field-effect transistors (MOSFETs). In particular, TFETs can have an SS lower than 60 mV/dec, which cannot be achieved by the conventional MOSFETs at room temperature because of their operation mechanism [4,5,6,7]. However, the conventional silicon-based TFETs exhibit several critical problems, in particular, low on-state current (*I*_on_). Due to the large bandgap (*E*_g_) of Si, the amount of electron band-to-band tunneling (BTBT) is insufficient, thus resulting in a small *I*_on_ [8,9,10,11]. Therefore, various studies have been conducted to improve these problems in material or structural approaches. TFETs using small *E*_g_ materials, such as Ge, at the source region exhibit an improvement in the amount of source-to-channel BTBT [12]. Furthermore, III–V heterojunction TFETs have been investigated for enhancing the electrical properties [13,14,15]. In addition to those experiments, many attempts have been performed in structural approaches to overcome the drawbacks, such as line TFET, U-gate TFET, T-shaped TFET, L-shaped TFET, and vertical nanowire TFET (VNWTFET) [15,16,17,18,19,20,21,22,23,24]. However, it is still necessary to study TFETs having superior performances and a small size.

In this work, a Ge-based gate-metal-core VNWTFET has been optimally designed and analyzed using the technology computer-aided design (TCAD) simulations. By using the gate-metal-core structure, the proposed device has a wider BTBT junction and, thus, higher current drivability can be obtained at the same size as that of the conventional VNWTFETs. Direct current (DC) characteristics such as *I*_on_, *I*_off_, the on–off current ratio (*I*_on_/*I*_off_), threshold voltage (*V*_t_), and SS are investigated to evaluate the device performance. Moreover, several device parameters were modulated to obtain the optimized design values.

## 2. Device Structure and Description

**Figure 1:**
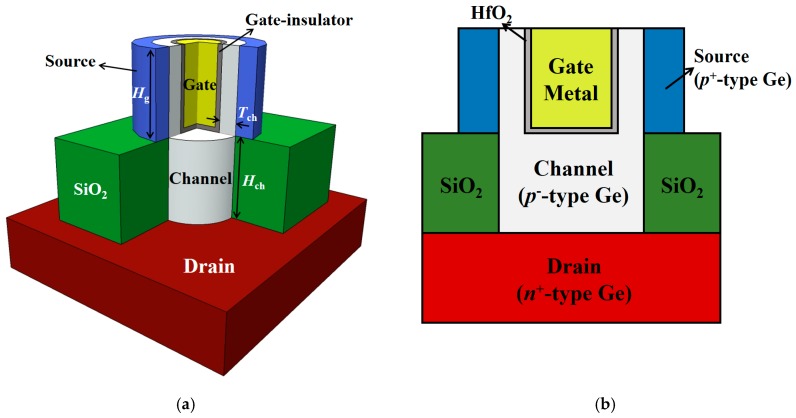
(**a**) Schematic view and (**b**) cross-sectional view of the proposed Ge-based gate-metal-core vertical nanowire tunnel field effect transistor (VNWTFET), respectively.

Figure 1 shows the cross-sectional view of the proposed Ge-based gate-metal-core VNWTFET with a gate radius (*R*_g_) of 10 nm and a gate dielectric thickness (*T*_ox_) of 2 nm. The gate dielectric material is hafnium oxide (HfO_2_), which enhances the current performances because of a higher gate controllability. The lower *E*_g_ and lower electron effective mass (*m*_e_^*^) of Ge can increase the BTBT rate [12]. The work function of the gate metal is 4.27 eV. The doping concentrations of the source, channel, and drain are *p*-type 1 × 10^20^ cm^−3^, *p*-type 1 × 10^16^ cm^−3^, and *n*-type 5 × 10^18^ cm^−3^, respectively. *I*_on_ is defined as the drain current (*I*_DS_) at the gate voltage (*V*_GS_) = the drain voltage (*V*_DS_) = 0.55 V, for low-power applications. Further, the threshold voltage (*V*_t_) is extracted using a constant-current method [25].

**Figure 2:**
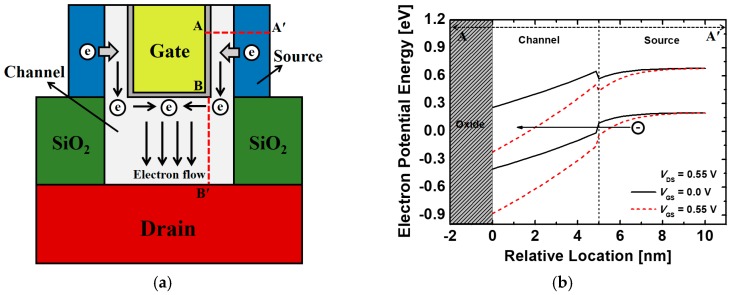
(**a**) The electron flow and (**b**) energy band diagrams of the proposed Ge-based gate-metal-core VNWTFETs. Energy band diagrams are extracted across the A–A’ line in Figure 2a.

Figure 2 shows the mechanism of the current flow in the proposed device. As indicated in Figure 2a, the electrons are tunneled mainly from the source to the channel regions in the lateral path and the tunneled electrons drift toward the drain region by *V*_DS_. When the positive *V*_GS_ is applied, the energy bands in the channel region are lowered and BTBT occurs at the channel–source interfaces as shown in Figure 2b. Therefore, the channel thickness (*T*_ch_) and the gate-metal height (*H*_g_) were considered as design variables for optimization processes because *T*_ch_ and *H*_g_ determine the tunneling probability and current drivability. Furthermore, the proposed gate-metal-core structure has the advantage that it proposes a wider source–channel junction area (A = 2π × (*R*_g_ + *T*_ox_ + *T*_ch_) × *H*_g_) than the conventional core–shell structure (A = 2π × *T*_ch_ × *H*_g_) in the same dimensions. Additionally, in the case of TFETs, a short source-to-drain distance causes the leakage current at the off-state and the ambipolar behavior when the negative *V*_GS_ is applied. Thus, the channel height (*H*_ch_) was also considered as a design parameter. The silicon dioxide (SiO_2_) is placed between the source and drain regions to suppress the leakage current.

The device design and analysis are performed with the Sentaurus TCAD simulation. During the simulation process, various physical models were included for the higher accuracy. A nonlocal BTBT model was applied because the drive current of the proposed device is totally affected by the amount of tunneled electrons. The generation rate (*R*_net_) by the nonlocal BTBT mechanism can be obtained by the follow equation:

(1) $$R_{\mathrm{net}}=A{\left(\frac{F}{F_{0}}\right)}^{P}\exp\left(-\frac{B}{F}\right)$$

where *F*_0_ = 1 V/cm, *P* = 2.5 for the phonon-assisted tunneling process. At *T* = 300 K, the prefactor, *A,* and the exponential factor, *B,* for the phonon-assisted tunneling process can be expressed by the follow equations:

(2) $$A=\frac{g{\left(m_{c}m_{v}\right)}^{3/2}\left(1+2N_{\mathrm{op}}\right)D^{2}{}_{\mathrm{op}}{\left(qF_{0}\right)}^{5/2}}{2^{21/4}h^{5/2}m_{r}^{5/4}\rho \varepsilon_{\mathrm{op}}E_{g}^{7/4}}$$

(3) $$B=\frac{2^{7/2}\pi m_{r}^{1/2}E_{g}^{3/2}}{3qh}$$

where *g* is a degeneracy factor, h is Plank’s constant, and *D*_op_, *ε*_op_, and *N*_op_ are the deformation potential, energy, and number of optical phonons, respectively. *ρ* is the mass density. *m*_C_ and *m*_V_ are the effective mass in the conduction band and the valance band, respectively, with the relationship of $\frac{1}{m_{r}}=\frac{1}{m_{V}}+\frac{1}{m_{C}}$. According to the Equations (1)–(3), the proposed Ge-based TFET can achieve the higher *R*_net_ due to the low *m*_e_^*^ and the low *E*_g_. The Fermi–Dirac statistical model was applied because the electrons in thermal equilibrium with a semiconductor lattice obey Fermi–Dirac statistics. In addition, the Shockley–Read–Hall (SRH) recombination model, auger recombination model, and trap-assisted-tunneling (TAT) model were involved because the recombination/generation, which influences the leakage current in the device, is greatly affected by the SRH and TAT mechanism. Moreover, the bandgap narrowing model, doping dependent mobility model, and quantum confinement effect were considered to estimate the device performances more accurately [26].

## 3. Results and Discussion

**Figure 3:**
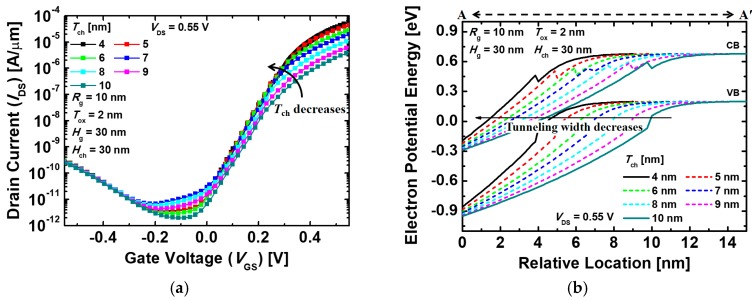
(**a**) *I*_DS_–*V*_GS_ (drain current–gate voltage) transfer characteristics and (**b**) energy band diagrams of the proposed devices with different channel thicknesses (*T*_ch_). The energy band diagrams are extracted across the A–A’ line in Figure 2a.

**Figure 4:**
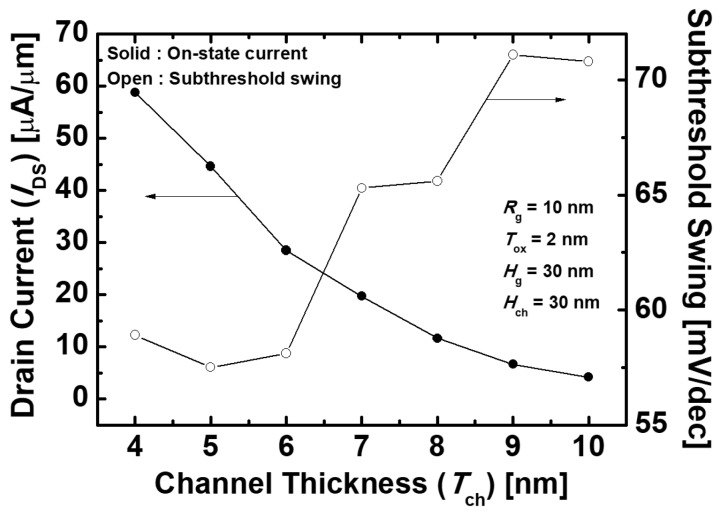
*I*_on_ and subthreshold swing (SS) characteristics of the proposed Ge-based gate-metal-core VNWTFETs with different *T*_ch_.

Figure 3a shows the *I*_DS_–*V*_GS_ transfer characteristics of the proposed gate-metal-core VNWTFETs that vary with different *T*_ch_. As *T*_ch_ gets thinner, *I*_on_ increases since the effective tunneling barrier width decreases. Figure 3b depicts the energy band diagrams of the proposed devices with different *T*_ch_. The electric field across the channel region also gets stronger as *T*_ch_ decreases, resulting in the enhancement of the gate controllability. Thus, the thinner *T*_ch_, having an energy band with a sharp slope, results in an increase of the electron tunneling rate. Moreover, *I*_off_ also increases as *T*_ch_ becomes thinner. When *T*_ch_ is 6 nm, however, *I*_off_ decreases because of the increment of the resistance of the channel (*R*_ch_), and then *I*_off_ increases again as *T*_ch_ further decreases. Figure 4 indicates the *I*_on_ and *SS* characteristics of the proposed devices with the different *T*_ch_. As described earlier, it is shown that *I*_on_ increases as *T*_ch_ reduces. Unlike *I*_on_, however, *SS* improved until *T*_ch_ becomes 5 nm, having the minimum value of 57.5 mV/dec, and thereafter increases because of the increment of *I*_off_. On the other hand, the ambipolar behavior was scarcely affected by *T*_ch_, since *H*_ch_, which contributes to the leakage current when the negative *V*_GS_ being applied is constant. Since *SS* is as crucial as *I*_on_ in the performance of the logic devices, it is desirable that *T*_ch_ is adjusted to be 5 nm. Consequently, *I*_on_ = 4.46 × 10^−5^ A/μm, *I*_off_ = 1.35 × 10^−11^ A/μm, *V*_t_ = 0.24 V, *I*_on_/*I*_off_ = 3.3 × 10^6^, and *SS* = 57.5 mV/dec are obtained at *T*_ch_ = 5 nm.

**Figure 5:**
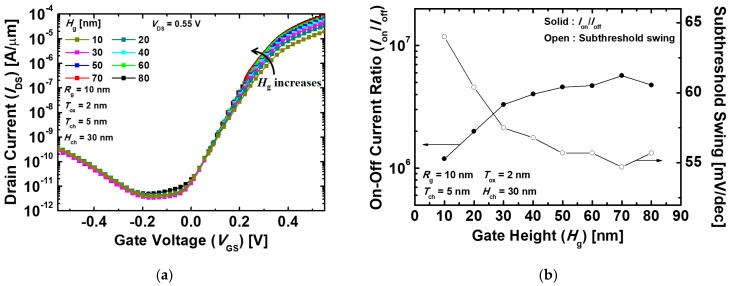
(**a**) *I*_DS_–*V*_GS_ transfer characteristics and (**b**) *I*_on_/*I*_off_ (on-state current/off-state current) and SS of the proposed Ge-based gate-metal-core VNWTFETs with different gate-metal heights (*H*_g_).

Figure 5a shows the *I*_DS_–*V*_GS_ transfer characteristics of the proposed devices according to variation in *H*_g_. Each curve was extracted from the devices with the different *H*_g_ varying from 10 to 80 nm at *T*_ch_ = 5 nm. The higher *H*_g_ widens the tunneling area, resulting in the enhancement of *I*_on_ because the *I*_DS_ of TFETs is totally affected by the amount of the tunneled electrons. Meanwhile, *I*_off_ also tends to increase slightly with the higher *H*_g_ for the same reason mentioned above. However, when *H*_g_ = 30 nm and *H*_g_ = 70 nm, *I*_off_ decreased because the increment of *R*_ch_, which resulted from the longer current path, dominates over the increase of the amount of the electron tunneling at the off-state. Furthermore, the increase of *R*_ch_ deteriorates the rate of the *I*_on_ increment, thus, *I*_on_ is gradually saturated. For these reasons, *I*_on_/*I*_off_ and SS have the largest value and the lowest value at *H*_g_ = 70 nm, respectively, as indicated in Figure 5b. Therefore, optimized values were obtained with *I*_on_ = 8.22 × 10^−5^ A/μm, *I*_off_ = 1.45 × 10^−11^ A/μm, *V*_t_ = 0.21 V, *I*_on_/*I*_off_ = 5.67 × 10^6^, and SS = 54.7 mV/dec at *T*_ch_ = 5 nm and *H*_g_ = 70 nm.

**Figure 6:**
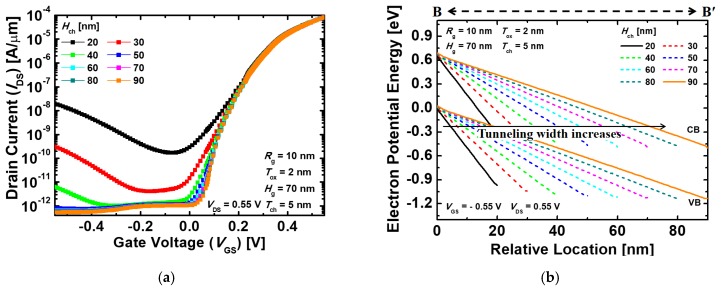
(**a**) *I*_DS_–*V*_GS_ transfer characteristics and (**b**) energy band diagrams of the proposed Ge-based gate-metal-core VNWTFETs with different channel heights (*H*_ch_). The energy band diagrams are extracted across the B–B’ line in Figure 2a at *V*_GS_ = −0.55 V and *V*_DS_ = 0.55 V (drain voltage).

**Figure 7:**
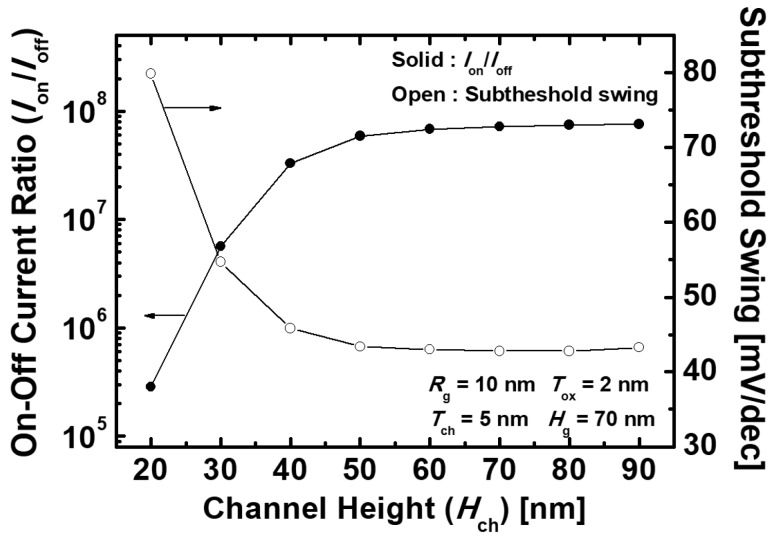
*I*_on_ and SS characteristics of the proposed Ge-based gate-metal-core VNWTFETs with different *H*_ch_.

Figure 6a shows the *I*_DS_–*V*_GS_ transfer characteristics of the proposed devices that vary with *H*_ch_. In the proposed device, the ambipolar behavior, when the negative *V*_GS_ is applied, is mainly affected by the amount of the electron tunneling from the source and channel region to the drain region. Figure 6b depicts the energy band diagrams of the proposed devices that vary with *H*_ch_ at *V*_GS_ = −0.55 V and *V*_DS_ = 0.55 V. With the lower *H*_ch_, the energy band of the channel is lowered by *V*_DS_, as in the conventional short channel TFETs [27]. Therefore, the longer *H*_ch_ where *V*_DS_ has less effect on the channel has the thicker tunneling barrier width at the channel–drain junction and, thus, suppresses the electron tunneling from the channel to the drain. In addition to the foregoing, as *H*_ch_ increases, *R*_ch_ increases because the current path also becomes longer. As a result, *I*_off_ decreases gradually with the *H*_ch_ increasing. The increase in *R*_ch_ also deteriorates *I*_on_ for the same reason. Figure 7 indicates *I*_on_/*I*_off_ and SS characteristics of the proposed devices with the different *H*_ch_ varying from 20 to 90 nm. *I*_on_/*I*_off_ is gradually improved as *H*_ch_ increases, and is then almost saturated when *H*_ch_ = 80 nm. Moreover, SS has minimum values at *H*_ch_ = 80 nm and then increases at *H*_ch_ = 90 nm. As mentioned above, because of the effect of *R*_ch_, *I*_on_ and *I*_off_ decrease constantly with increasing *H*_ch_, and the decrease in *I*_on_ dominates over *I*_off_ when *H*_ch_ = 80 nm. Finally, the optimized device is achieved with *I*_on_ = 8.09 × 10^−5^ A/μm, *I*_off_ = 1.09 × 10^−12^ A/μm, *V*_t_ = 0.21 V, *I*_on_/*I*_off_ = 7.45 × 10^7^, and SS = 42.8 mV/dec at *T*_ch_ = 5 nm, *H*_g_ = 70 nm, and *H*_ch_ = 80 nm.

**Figure 8:**
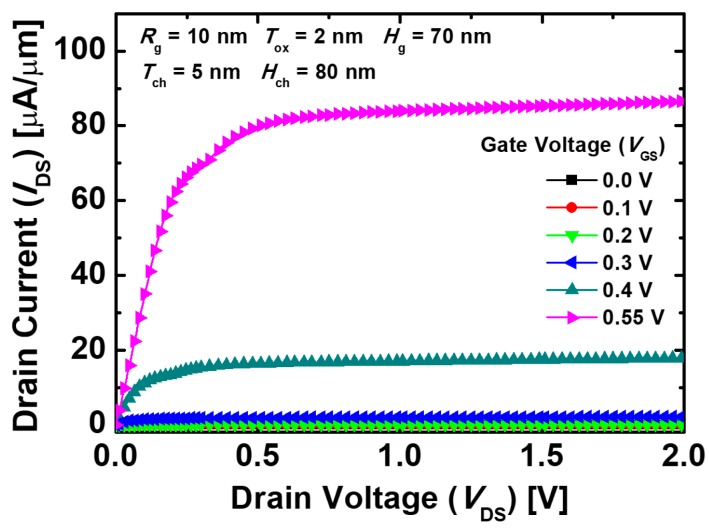
Output characteristics of the proposed Ge-based gate-metal-core VNWTFETs with different *V*_GS_.

Figure 8 indicates the output characteristics of the proposed devices with different *V*_GS_. When a small *V*_GS_ of 0.3 V or less is applied, *I*_DS_ is almost constant and has a low value. *I*_DS_ has a low value even though *V*_DS_ increases because the amount of the electrons tunneled from the source to the channel region is small for a low *V*_GS_. When *V*_GS_ is greater than 0.4 V, the tunneled electrons in the channel region drift toward the drain region by the positive *V*_DS_. *I*_DS_ increases as a function of *V*_DS_ for small *V*_DS_, then gets saturated and becomes less dependent on *V*_DS_ when *V*_DS_ is approximately 0.5 V, showing the proper output characteristics for circuit applications.

The comparison of the proposed device with the different works in terms of *I*_on_, *V*_t_, *V*_DD_, and SS is presented in Table 1. This proposed device has the highest *I*_on_ value with the low *V*_t_ and the low *V*_DD_ value. Thus, the proposed Ge-based gate-metal-core VNWTFET is a suitable candidate for the logic devices with the low power consumption.

## 4. Conclusions

In this work, a Ge-based gate-metal-core VNWTFET was optimally designed and analyzed based on TCAD simulations. With wider BTBT junctions, a higher current drivability can be realized compared to the conventional TFETs. The proposed device demonstrated superior DC performances with *I*_on_ = 8.09 × 10^−5^ A/μm, *I*_off_ = 1.09 × 10^−12^ A/μm, *V*_t_ = 0.21 V, *I*_on_/*I*_off_ = 7.45 × 10^7^, and SS = 42.8 mV/dec at *H*_g_ = 70 nm, *T*_ch_ = 5 nm, and *H*_ch_ = 80 nm. It is ensured that the proposed device would be a promising logic device for the low-power applications.

## Figures and Tables

**Table 1:** Comparison with the different works.

Parameter	This Work	SiGe-S-NW-TFET [28]	Si-Based Nanotube TFET [29]	Si/SiGe HTG-TFET [30]	Ge-Source vTFET [31]
*I*_on_ (μA/μm)	80.9 (at *V*_GS_ = 0.55 V)	11.66 (at *V*_GS_ = 1.0 V)	5.0 (at *V*_GS_ = 1.5 V)	7.02 (at *V*_GS_ = 0.5 V)	27.6 (at *V*_GS_ = 0.5 V)
*V*_t_ (V)	0.21	0.37	0.9	0.28	0.20
*V*_DD_ (V)	0.55	0.8	1.2	0.5	0.5
SS (mV/dec)	42.8	23.75	58.3	44.64	21.2
